# Targeting aspartate aminotransferase in breast cancer

**DOI:** 10.1186/bcr2154

**Published:** 2008-10-15

**Authors:** Joshua Marshall Thornburg, Kristin K Nelson, Brian F Clem, Andrew N Lane, Sengodagounder Arumugam, Allan Simmons, John W Eaton, Sucheta Telang, Jason Chesney

**Affiliations:** 1 Department of Medicine (Medical Oncology), J.G. Brown Cancer Center, University of Louisville, 580 South Preston Street, Delia Baxter II Building, Room 211, Louisville, KY 40202, USA

## Abstract

**Introduction:**

Glycolysis is increased in breast adenocarcinoma cells relative to adjacent normal cells in order to produce the ATP and anabolic precursors required for survival, growth and invasion. Glycolysis also serves as a key source of the reduced form of cytoplasmic nicotinamide adenine dinucleotide (NADH) necessary for the shuttling of electrons into mitochondria for electron transport. Lactate dehydrogenase (LDH) regulates glycolytic flux by converting pyruvate to lactate and has been found to be highly expressed in breast tumours. Aspartate aminotransferase (AAT) functions in tandem with malate dehydrogenase to transfer electrons from NADH across the inner mitochondrial membrane. Oxamate is an inhibitor of both LDH and AAT, and we hypothesised that oxamate may disrupt the metabolism and growth of breast adenocarcinoma cells.

**Methods:**

We examined the effects of oxamate and the AAT inhibitor amino oxyacetate (AOA) on ^13^C-glucose utilisation, oxygen consumption, NADH and ATP in MDA-MB-231 cells. We then determined the effects of oxamate and AOA on normal human mammary epithelial cells and MDA-MB-231 breast adenocarcinoma cell proliferation, and on the growth of MDA-MB-231 cells as tumours in athymic BALB/c female mice. We ectopically expressed AAT in MDA-MB-231 cells and examined the consequences on the cytostatic effects of oxamate. Finally, we examined the effect of AAT-specific siRNA transfection on MDA-MB-231 cell proliferation.

**Results:**

We found that oxamate did not attenuate cellular lactate production as predicted by its LDH inhibitory activity, but did have an anti-metabolic effect that was similar to AAT inhibition with AOA. Specifically, we found that oxamate and AOA decreased the flux of ^13^C-glucose-derived carbons into glutamate and uridine, both products of the mitochondrial tricarboxylic acid cycle, as well as oxygen consumption, a measure of electron transport chain activity. Oxamate and AOA also selectively suppressed the proliferation of MDA-MB-231 cells relative to normal human mammary epithelial cells and decreased the growth of MDA-MB-231 breast tumours in athymic mice. Importantly, we found that ectopic expression of AAT in MDA-MB-231 cells conferred resistance to the anti-proliferative effects of oxamate and that siRNA silencing of AAT decreased MDA-MB-231 cell proliferation.

**Conclusions:**

We conclude that AAT may be a valid molecular target for the development of anti-neoplastic agents.

## Introduction

Increased uptake of glucose as an anaerobic source of energy and biosynthetic precursor is a common feature of primary and metastatic breast adenocarcinomas. Positron-emission tomographic (PET) studies with 2-[^18^F]fluoro-2-deoxy-glucose have consistently demonstrated that human breast tumours transport increased glucose relative to adjacent normal breast tissues *in vivo *[1]. Understanding the regulation of glucose uptake and metabolism in breast cancer may therefore provide new metabolic targets for the development of anti-breast cancer agents.

The proto-oncogene c-myc is amplified in breast tumours and is associated with an increased risk of relapse and death [2]. C-myc induces increased glycolytic flux in cancer cells partly through transcriptional promotion of lactate dehydrogenase (LDH) A, a glycolytic enzyme required for c-myc-driven growth [3]. LDH-A is a reversible enzyme that converts pyruvate to lactate and oxidises the reduced form of nicotinamide adenine dinucleotide (NADH) to NAD+ [4,5]. LDH-A expression and activity is increased in malignant relative to normal breast tissues [6,7]. LDH-A is also upregulated by the transcription factor, hypoxia inducible factor 1α (HIF-1), which is a central regulator of the metabolic response to hypoxia. HIF-1α is over-expressed in breast tumours and is positively associated with proliferation and therefore poor outcome in breast cancer [8,9]. Finally, LDH activity is increased by oestrogen and oestrogen receptor-positive breast tumours express increased LDH-A levels relative to adjacent normal tissues [10,11]. Taken together, these data suggest that LDH may be a rational target for the development of agents that disrupt the glycolytic metabolism of breast cancer cells.

LDH is composed of two separately encoded subunits, A and B, that combine to form binomial distributions of the five expected LDH tetramers: LDH1-B_4 _(also known as LDH-B), LDH2-B_3_A, LDH3-B_2_A_2_, LDH4-BA_3 _and LDH5-A_4 _(also known as LDH-A) [12]. Oxamate is an established pyruvate analogue and competitive inhibitor of LDH-A [13-16]. Oxamate also inhibits aspartate aminotransferase (AAT), which functions in tandem with malate dehydrogenase to form the malate-aspartate NADH shuttle. AAT is required to shuttle electrons from glycolysis-derived cytoplasmic NADH to mitochondrial NADH, which are then used by the electron transport chain to generate a proton gradient necessary for oxidative phosphorylation [17-19]. Importantly, oxamate suppresses the glycolytic flux and growth of HeLa cervical adenocarcinoma cells [16] and is selectively toxic to fibroblasts transformed with H-ras [20], an oncogene that is over-expressed in 50 to 70% of breast adenocarcinomas relative to adjacent normal breast tissues [21-25]. Given the high activity of LDH in breast tumours and the potential role of AAT in NADH shuttling into the mitochondria, we postulated that oxamate, which inhibits both LDH and AAT, may suppress the growth of human breast adenocarcinomas.

In the current study, we confirm that oxamate has anti-growth effects on breast cancer cells *in vitro *and *in vivo *but find that the cytostatic effects are secondary to inhibitory effects on mitochondrial metabolism rather than glycolysis. In particular, we identify AAT as an essential *in situ *metabolic target of oxamate and demonstrate that selective inhibition of AAT with either a carbonyl-trapping agent, amino oxyacetate (AOA) or AAT-specific siRNA species decreases the proliferation of breast adenocarcinoma cells. We conclude that AAT may be essential for the transformation and growth of mammary epithelial cells and may therefore prove to be a valid target for the development of anti-breast cancer agents.

## Materials and methods

### Cloning human LDH-A and AAT protein

LDH-A and AAT cDNA were amplified from normal human epithelial cell cDNA using PCR primers designed to flank the open reading frame. The PCR product was then subcloned into a pET-30b(+) vector (Novagen, San Diego, CA). BL21(DE3) *Escherichia coli *competent cells (Novagen, San Diego, CA) were transformed with the pET-30b(+)-LDHA and pET-30b(+)-AAT, C-termHis plasmids. For expression and purification of protein, 250 mL cultures of BL21-LDHA and BL21-AAT transformed cells were incubated for 16 hours at 37°C and 250 mL of Luria-Bertani media containing 2 mM isopropyl β-D-1-thiogalactopyranoside (final concentration = 1 mM) was added to each culture and shaken for an additional four hours at 30°C. Bacteria were collected by centrifugation, and protein purification was performed as described in the Qiaexpressionist protocol (Qiagen Valencia, CA). Protein concentration in elution fractions was quantified using a bicinchoninic acid protein assay (Pierce Biotechnologies, Rockford, IL) according to the manufacturer's protocol for both LDH-A and AAT protein products.

### LDH-A assay

The effect of oxamate on LDH-A was measured using purified human enzyme. In 300 μL of 100 mM sodium phosphate buffer (pH 7.5), 0.025 to 0.075 units of LDH-A was combined with reduced β-NADH, 0 to 3 mM sodium pyruvate and 0.033% BSA. Absorbance at 340 nm was measured using a 96-well plate reader to determine the maximum linear change of absorbance at 340 nm/minute as a measurement of enzyme activity. LDH activity in the presence of several concentrations of oxamate was measured and analysed using Sigma Plot Enzyme Kinetics Module (Systat Software, Richmond, CA).

### AAT assay

The effect of oxamate on AAT was measured using purified human recombinant enzyme. In a 300 μL reaction, 193 mM L-aspartate, 12 mM α-ketoglutarate, 0.6 units/mL malate dehydrogenase, 0.2 mM NAD and 0.03 to 0.06 unit human recombinant AAT were combined. Absorbance at 340 nm was measured using a 96-well plate reader to determine the maximum linear change of absorbance as a measurement of enzyme activity. AAT activity, in the presence of several concentrations of oxamate was measured to determine *in vitro *AAT inhibition. Enzyme activity was analysed using Sigma Plot Enzyme Kinetics Module (Systat Software, Richmond, CA).

### Cell culture

MDA-MB-231 breast adenocarcinoma cells (HTB-26, American Type Culture Collection Manassas, VA) were maintained in Dulbecco's Modified Eagle's Medium (DMEM) supplemented with 10% FCS and gentamicin sulfate (HyClone Logan, UT). Normal human mammary epithelial cells (HMEC; Lonza, CC-2551, Walkersville, MD) were maintained in media formulated with bovine pituitary extract, human epidermal growth factor, hydrocortisone, GA-1000 and insulin (Lonza, Walkersville, MD).

### *In vitro *cell proliferation assay

MDA-MB-231 cells and HMEC cells were cultured for 72 hours in identical growth factor-enriched media formulated with bovine pituitary extract, human epidermal growth factor, hydrocortisone, GA-1000 and insulin. Several concentrations of sodium oxamate or AOA (Sigma, St. Louis, MO) were then added to the media for 48 hours and viable cells were enumerated by light microscopy using trypan blue to exclude dead cells. The media contained glucose (5 mM) and glutamine (1 mM), and the cells were cultured under 21% oxygen (normoxia) with 5% carbon dioxide at 37°C using a variable carbon dioxide tension incubator (Heraeus, Thermo Fisher Scientific, Asheville, NC). For routine passage, cells were washed with PBS and trypsinised. Because cultured cells can undergo mutations, frozen stocks were maintained and active cultures were restarted from these stocks every 30 days.

### Soft agar colony formation assays

MDA-MB-231 cells were plated at a density of 25 × 10^3 ^cells per 60-mm plate with 3 ml bottom agar (0.6%) and 2 ml top agar (0.3%). HMEC cells served as a negative control, because they do not grow in soft agar. Cells were fed every five days by adding a new layer of top agar containing the indicated concentration of oxamate or AOA. After colonies reached a minimum of 50 μm, they were counted in random 1 cm squared sections of each plate.

### MDA-MB-231 xenografts in athymic mice

MDA-MB-231 cells were resuspended in 100 μL PBS and mixed with 100 μL cold Matrigel basement membrane matrix (BD Biosciences, Bedford, MA). The resultant mixture (200 μL containing 10 × 10^6 ^cells) was injected subcutaneously into the flanks of groups of 10 BALB/c nude female mice (average weight 20 g) per experimental group. When xenografts reached a mass of 120 mg, a subset of mice received an intraperitoneal injection with 15 mg oxamate or 0.2 mg AOA, every 24 hours for 14 days. The mass of the resultant tumours was determined every 24 hours with vernier calipers according to the following formula: mass (mg) = [width (mm)]^2 ^× [length (mm)]/2 [26,27].

These studies followed internationally recognised guidelines and were approved by the University of Louisville Institutional Animal Care and Use Committee (IACUC #06007).

### Lactate measurements

Lactate concentrations in the media were measured using a lactate oxidase-based assay read at 540 nm (Trinity, Wicklow, Ireland). Lactate data were normalised to cell number. Experiments were repeated three times and are expressed as mean ± standard deviation.

### Nuclear magnetic resonance

We examined the effect of oxamate or AOA on the flux of glucose carbons into anabolic pathways by capturing 2D nuclear magnetic resonance (NMR) spectra of MDA-MB-231 cells treated with oxamate (40 mM) or AOA (100 μM) for 48 hours in the presence of uniformly labelled ^13^C-glucose (1 gm/L) (in glucose-free DMEM, 10% dialysed FCS). For 24 hours, 4 × 10^6 ^MDA-MB-231 cells were plated in T75 cell culture flasks, then washed with PBS and the media was replaced with DMEM containing 5 mM [U-^13^C_6_]-glucose, 10% dialysed FCS and gentamicin. Cells were cultured with or without 40 mM oxamate or 100 μM AOA (5% carbon dioxide, 37°C for 96 hours), trypsinised and cell pellets were washed with PBS.

Cell pellets and supernatants were flash frozen in liquid nitrogen, and then ground frozen in liquid nitrogen. The pulverised samples were lyophilised (5 to 20 mg) and extracted with 20 to 30:1 (v/w) (dried) volumes of 10% (v/v) trichloracetic acid (TCA), respectively, on ice, before centrifugation. The mixture was centrifuged (4°C) at 15,000 rpm in an SA-600 rotor (Sorvall, DuPont, Wilmington, DE) for 30 minutes, and the supernatant was removed and saved. The residue was extracted again, centrifuged and the supernatants were pooled.

Two aliquots (100 to 200 μl each) of the final extract were lyophilised in 1.5 ml Eppendorf tubes. Dried extract samples were redissolved in 100% heavy water, centrifuged to remove particulates and loaded into appropriate NMR tubes. DSS (2,2-dimethyl-2-silapentane-5-sulfonate sodium) was added as an internal chemical shift reference and concentration standard. NMR spectra were recorded at 14.1 T on Varian Inova NMR spectrometer (Varian, Palo Alto, CA) at 20°C using a 90° excitation pulse. Total correlation spectroscopy (TOCSY) experiments used a 6000 Hz spectral width both dimensions, 0.341 second acquisition time in t2 and 0.05 second in t1, a recycle time of 1.84 seconds, a 50 ms mixing time, and a B1 field strength of 8 kHz generated with MLEV-17. Metabolites were identified based on their 1H and 13C chemical shifts, and TOCSY connectivity pattern.

### Oxygen consumption

MDA-MB-231 cells were grown in oxamate (40 mM) or AOA (100 μM) for 24 hours. Subsequently, cells were detached with trypsin-ethylenediaminetetraacetic acid (EDTA), and centrifuged at 1000 rpm for five minutes in complete culture medium. Oxygen consumption was measured using a Strathkelvin 782 Oxygen Meter (Strathkelvin Instruments Ltd. Glasgow Scotland, UK). Respiration rates were measured using 5 × 10^6 ^cells suspended in a total volume of 525 μL DMEM containing 10% FBS at 37°C for 10 minutes.

### Cellular ATP determination

Cellular ATP was measured using a bioluminescence assay determination kit (Molecular Probes, Invitrogen, Carlsbad, CA.) The assay relies on the requirement of ATP by luciferase to produce light (emission maximum about 560 nm at pH 7.8). Briefly, after 24 hours of growth in oxamate (40 mM) or AOA (100 μM), 1 × 10^6 ^cells were resuspended in reaction buffer containing 1 mM dithiothreitol (DTT), 0.5 mM luciferin and 12.5 μg/ml luciferase, gently mixed, and measurements were obtained in a luminometer (TD-20/20, Turner Designs, Sunnyvale, CA). ATP standard curves were run concurrently with each experiment to produce a standard curve, and calculations were made against the curve to determine cellular ATP levels (expressed as mol/10^6 ^cells).

### NAD+/NADH ratio assay

MDA-MB-231 cells were grown to 70% confluency in a 10 cm^2 ^tissue culture plate in appropriate media (as described above). Cells were subsequently treated with media containing oxamate (40 mM) or AOA (100 μM). After 24 hours of treatment, cells were lifted with trypsin, washed twice with PBS and 1 × 10^6 ^cells were pelleted through centrifugation. NADH and NAD+ were determined according to manufacturer's protocol (#ECND-100, Bioassay Systems, Hayward, CA). Briefly, cell pellets were resuspended in 1.5 mL Eppendorf tubes with either 100 μl NAD+ extraction buffer (containing 0.40% hydrochloric acid) for NAD determination or 100 μl NADH extraction buffer (containing 0.40% sodium hydroxide) for NADH determination. Extracts were heated for five minutes at 60°C and 20 μl of assay buffer (containing Tris (hydroxymethyl) aminomethane 3.0% and BSA 0.10%) was added followed by the extraction buffer (to neutralise the extracts). Mixtures were vortexed and centrifuged at 13,000 rpm for five minutes. Supernatants were added to working reagent containing assay buffer, alcohol dehydrogenase, ethanol, phenazine methosulfate (PMS) and tetrazolium dye (MTT). Optical density at 565 nm was recorded at time zero and at 15 minutes using a 96-well plate reader spectrophotometer. Difference in absorbance was compared with standard solutions and used to calculate NADH and NAD+ concentrations.

### Ectopic expression of AAT

MDA-MB-231 cells were transfected with the open reading frame of the human cytoplasmic AAT using the pIRESneo3 vector (Clontech, Mountain View, CA), to create MDA-MB-231/AAT+ cells. MDA-MB-231 cells were also transfected with the empty pIRES/neo3 vector alone to create MDA-MB-231/vect cells as a negative control. This vector contains an internal ribosome entry site (IRES), thus permitting the translation of two open reading frames from one mRNA. After selection with 500 μg/ml neomycin, surviving cells stably expressed AAT (as confirmed with Western blot). All transfections were carried out using Lipofectamine (Invitrogen, Carlsbad, CA) as a transfection reagent. MDA-MB-231/AAT+ cells were cultivated in DMEM supplemented with 10% FCS and gentamycin sulfate (HyClone) containing 250 μg/ml neomycin.

### siRNA transfection

Cells were plated at a concentration of 65 × 10^3 ^cells per well in six-well tissue culture plates (or 1 × 10^6 ^cells/T150 flasks) and incubated in 5% carbon dioxide and 37°C in DMEM as described above for 24 hours. Cells were then transfected with siRNA for a final concentration of 100 nM using Oligofectamine (Invitrogen, Carlsbad, CA) as a transfection reagent according to the manufacturer's protocol. Three siRNA constructs were obtained from a predesigned siRNA library (Ambion, Applied Biosystems, Austin, TX), one targeting exon #4 of the GOT1 mRNA product (AAT1), one targeting exon #3 of the GOT1 mRNA product (AAT2) and one crossing exons #1 and 2 of the GOT1 mRNA product (AAT3). A nonspecific siRNA and Oligofectamine alone were included as negative controls. After transfection, cells were incubated for 48 hours where cells were counted and protein levels were quantified using Western blot analysis.

## Results

### Oxamate inhibits human recombinant LDH-A and AAT

**Figure 1:**
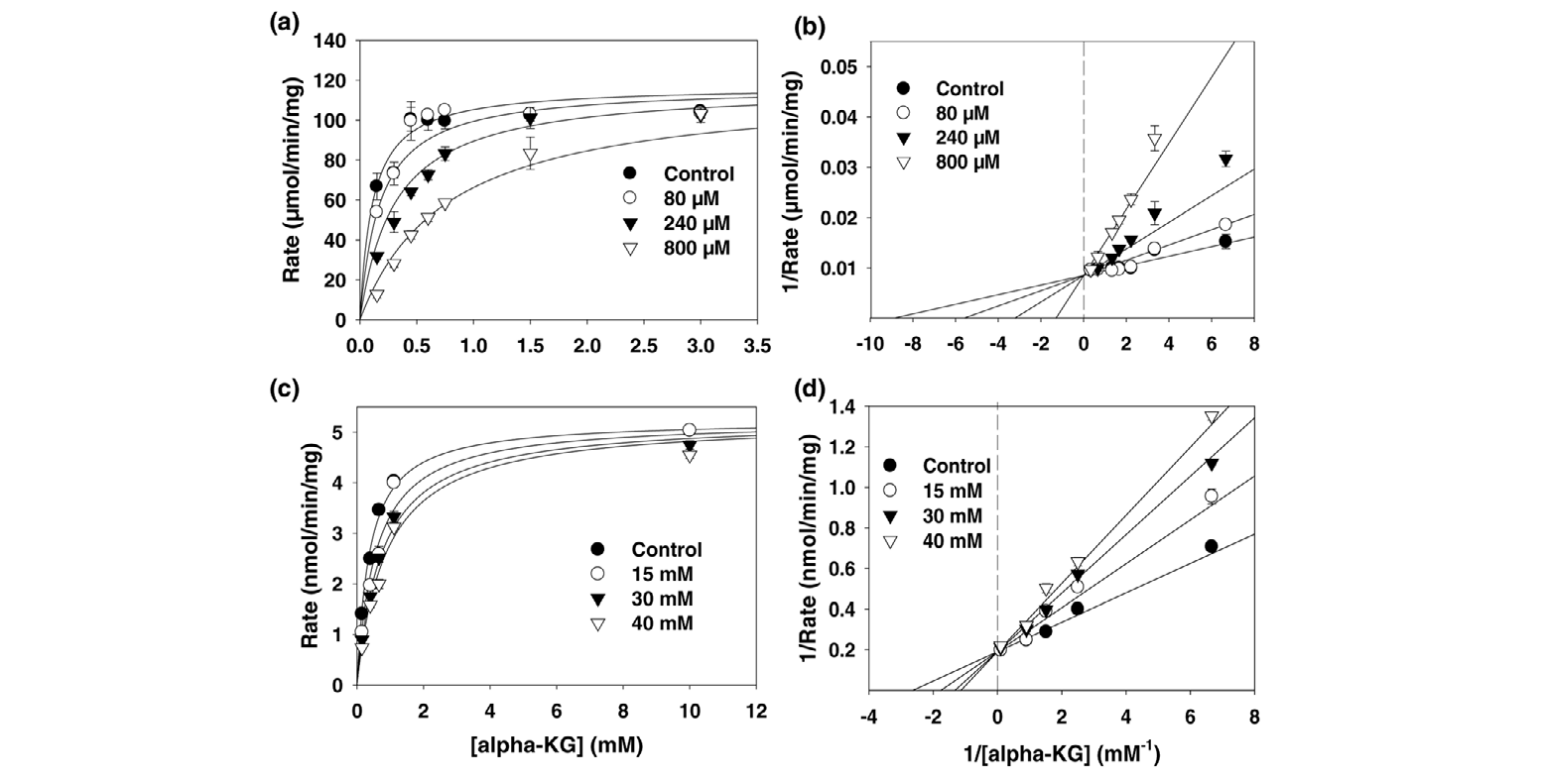
**Oxamate inhibits human recombinant lactate dehydrogenase A (LDH-A) and aspartate aminotransferase (AAT) activity *in vitro***. (a) Recombinant enzyme assays using purified human LDH-A were performed as described. (b) Lineweaver-Burk double reciprocal plots examining LDH-A enzyme activity as a function of pyruvate concentration. Kinase assays were performed in the presence or absence of 80, 240 or 800 μM oxamate. (c) *In vitro *recombinant enzyme assays using purified human AAT were performed as described. (d) Lineweaver-Burk double reciprocal plots examining AAT enzyme activity as a function of α-ketoglutarate (KG) concentration. Kinase assays were performed in the presence or absence of 15, 30 or 40 mM oxamate. Data are plotted as the mean ± standard deviation.

The enzyme kinetics of LDH and the inhibitory effects of oxamate on LDH using enzyme purified from rabbit muscle (LDH-A) or porcine heart (LDH-B) have been previously examined [19,27,28]. However, no studies have examined the effects of oxamate on purified human LDH-A and differences between the rabbit and human LDH-A amino acid sequences (77% homology) may result in differences in the affinity of oxamate. We produced recombinant human LDH-A and conducted initial kinetic studies that indicated that the Vmax of human LDH-A is 117 μmol/minute/mg protein and the Km for pyruvate is 111.8 μM (Figures 1a and 1b). We confirmed that oxamate competitively inhibits the activity of human LDH-A *in vitro*, with a Ki of 136.3 μM (using pyruvate as substrate). These data indicate that oxamate is a competitive inhibitor of human LDH-A and thus may prove useful for an examination of the effects of LDH-A inhibition on human breast adenocarcinoma cell proliferation.

Oxamate also has previously been found to inhibit purified AAT, an enzyme involved in mitochondrial metabolism via the tricarboxylic acid cycle and the malate-aspartate shuttle [17,28]. Specifically, oxamate can competitively inhibit AAT with respect to 2-oxoglutarate (α-ketoglutarate), having a Ki of 28 μM. We confirmed that oxamate inhibits the activity of recombinant human recombinant AAT *in vitro *(Vmax = 5.2 μmol/minute/mg protein, Km = 379.1 μM, Ki = 30.3 μM; Figure 1c). Lineweaver-Burk analyses indicated that the inhibitory effects were almost completely competitive with α-ketoglutarate (Figure 1d).

### Oxamate decreases the channeling of glucose-derived carbons into products of the tricarboxylic acid cycle

**Figure 2:**
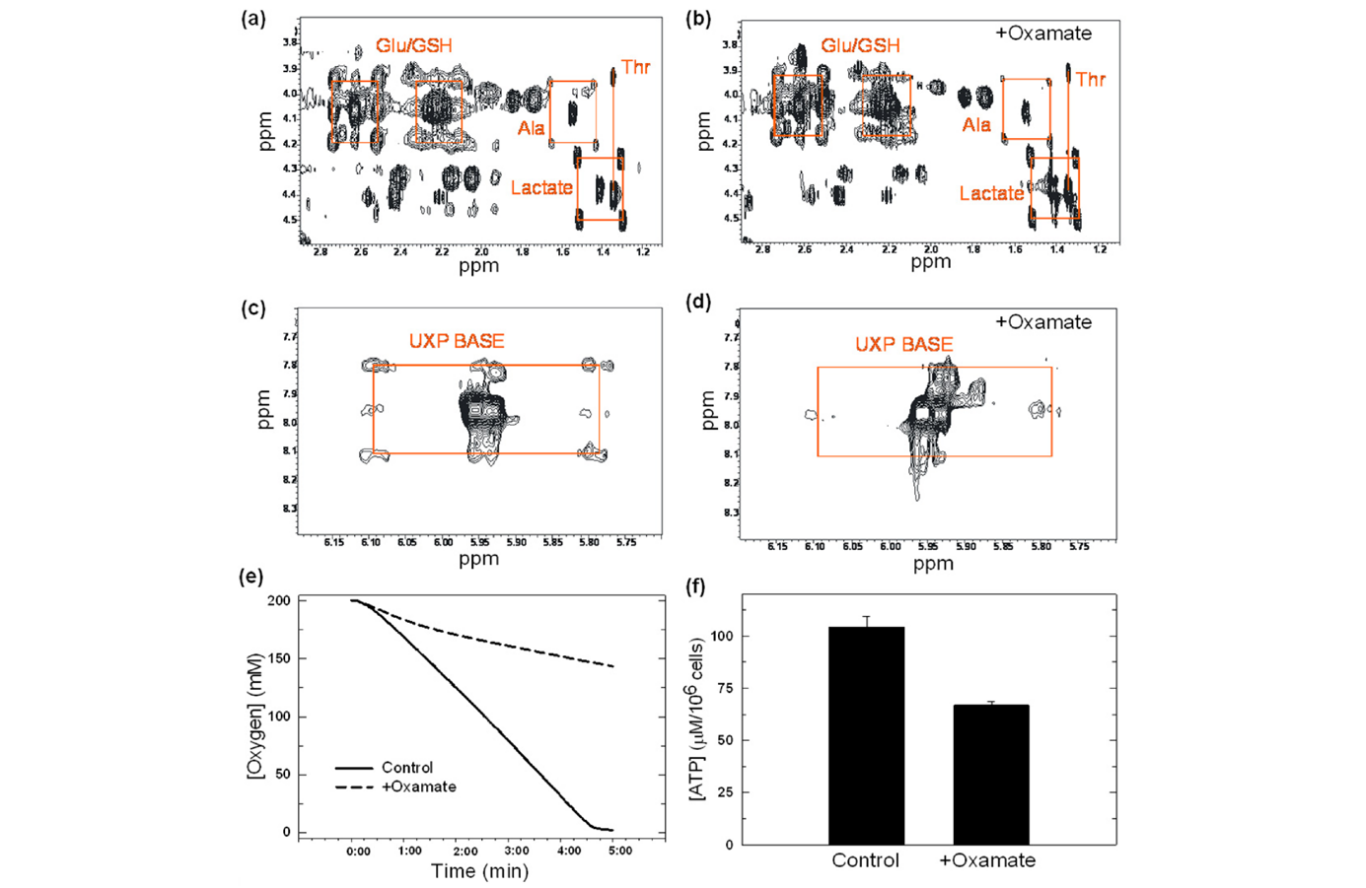
**Oxamate inhibits the conversion of glucose into several products of the tricarboxylic acid cycle, oxygen consumption and intracellular ATP**. MDA-MB-231 cells were grown in medium containing 1 g/L [U-^13^C_6_]-glucose and exposed to either (a, c) PBS or (b, d) 40 mM oxamate. After 48 hours, live cells were washed, extracted with 10% trichloracetic acid (TCA), lyophilised and dissolved in 100% heavy water. The spectrum was recorded at 14.1 T with a recycle time of five seconds under standardised conditions of acquisition. ^13^C-isotopomers were quantified by indirect detection of protons in the 2D-NMR total correlation spectroscopy (TOCSY) spectrum. The four peaks representing the ^13^C-labelled glutamate/glutathione (Glu/GSH), alanine (Ala) and (a, b) lactate and (c, d) uridine bases are highlighted with red squares. Cells were cultured in the presence or absence of 40 mM oxamate for 24 hours and then (e) oxygen consumption was measured using an oxygen meter and (f) ATP concentration was determined with a luminometer as detailed. ATP standard curves were run concurrently. Data are plotted as the mean ± standard deviation.

Given the sensitivity of human LDH-A to oxamate, we assumed that oxamate would reduce the cellular production of lactate. We examined the effect of oxamate on lactate secretion by MDA-MB-231 cells and, surprisingly, observed no difference in the lactate secreted per cell (control, 89 ± 11 mg/dL per 10^6 ^cells; +40 mM oxamate, 88 ± 14 mg/dL per 10^6 ^cells). In order to better clarify the metabolic consequences of oxamate on glycolytic flux, we pulsed MDA-MB-231 cells with uniformly-labelled [U-^13^C_6_]-glucose and examined the fate of the ^13^C using 2D-NMR-TOCSY analyses. Figures 2a and 2b show the regions of the TOCSY spectra of untreated and oxamate-treated MDA-MB-231 cells that contain several metabolites of interest, including glutamate/glutathione, lactate and alanine. Specifically, the cross peaks at 1.40/4.38 ppm represent ^12^C-lactate, while the satellite peaks (at the corners of the identifying squares) represent ^13^C-labelled lactate. We confirmed equal ^13^C-enrichment of intracellular lactate from the [U-^13^C_6_]-glucose in the untreated and oxamate-treated cells (compare Figures 2a and 2b). These data demonstrate that oxamate exposure to MDA-MB-231 cells is not causing significant inhibition of LDH-A activity *in situ*.

In Figures 2a and 2b, the peaks at 2.60/4.05 ppm and 2.20/4.05 ppm represent the glutamate/glutathione moieties with satellite peaks (again at the corners of the identifying squares) representing ^13^C-labelled glutamate/glutathione. We observed less ^13^C-labelled glutamate/glutathione in the oxamate-treated group (Figure 2b), which suggests that oxamate may decrease the conversion of glucose-derived ^13^C into glutamate. Glucose is converted to pyruvate via the glycolytic pathway, which is then either converted to lactate via LDH-A or funneled into the tricarboxylic acid cycle in the mitochondria. Through the tricarboxylic acid cycle, carbons deriving from pyruvate can be incorporated into several metabolites associated with the tricarboxylic acid cycle including glutamate. Based on the observations that there is less ^13^C-enrichment of glutamate in the oxamate-treated cells and that there is no difference in ^13^C incorporation into lactate, we surmise that oxamate may be suppressing the tricarboxylic acid cycle.

Figures 2c and 2d show the cross peak pattern centered at 8.00/5.95 ppm corresponding to the H6, H5 ring protons of uridine (UTP and UDP). The carbons of uridine are derived from aspartate, which is produced by the transamination of oxaloacetate from the tricarboxylic acid cycle. The satellite peaks in the untreated MDA-MB-231 cells (Figure 2c) indicate that the uridine base (UXP) incorporates carbons from normal tricarboxylic acid cycling in which the ^13^C-label enters as acetyl coenzyme A (via pyruvate dehydrogenase) and from anapleurotic reactions in which adjacent carbons from pyruvate are converted into oxaloacetate via pyruvate carboxylase. However, these satellite peaks are completely absent after oxamate treatment suggesting that the ^13^carbons are not being incorporated into uridine, and that normal tricarboxylic acid cycling in MDA-MB-231 cells is inhibited by oxamate treatment (Figure 2d).

We examined oxygen consumption, a direct measure of electron transport chain activity, by MDA-MB-231 cells with and without exposure to 40 mM oxamate for 24 hours (a period that precedes any cytostatic effects) and observed a 61% decrease in the rate of oxygen consumption (5 × 10^6 ^cells per group; Figure 2e). We also found that oxamate reduced the steady-state concentration of ATP after 24 hours (33 ± 3% reduction in ATP per cell; Figure 2f) and observed a small but statistically significant decrease in the NAD+/NADH ratio of MDA-MB-231 cells treated with 40 mM oxamate (untreated, NAD+ = 9.41 ± 0.63 μM, NADH = 7.02 ± 0.33 μM, NAD+/NADH = 1.34; + 40 mM oxamate, NAD+ = 9.68 ± 0.54 μM, NADH = 8.11 ± 0.33 μM, NAD+/NADH = 1.19). We suspect that this only limited decrease in the NAD+/NADH ratio may reflect compensation by the multitude of other enzymes that use NAD+/NADH as cofactors.

### AOA decreases the channeling of glucose-derived carbons into products of the tricarboxylic acid cycle

AAT is a pyridoxal phosphate-dependent enzyme that plays a major role in amino acid metabolism and the tricarboxylic acid cycle. When coupled to the malate dehydrogenase enzymes, AAT catalyses the activity of the malate-aspartate NADH shuttle, which faciliates the net transfer of reducing equivalents across the mitochondrial membrane [29]. Specifically, AAT is responsible for the interconversion of aspartate and glutamate with the co-conversion of α-ketoglutarate and oxaloacetate. Inhibition of AAT rather than LDH-A would be expected to reduce the conversion of glucose-derived carbons into glutamate and uridine, and to decrease oxygen consumption. Amino oxyacetate is a carbonyl-trapping agent that inhibits AAT because of its dependence on pyridoxal phosphate as a co-enzyme [30]. This inhibition is directly related to the concentration of AOA used [31]. Although the Ki value for AOA is not published, it has been previously identified as only a moderately potent inhibitor of AAT *in vivo*, requiring 1.0 mM to achieve sufficient inhibition [32]. However, more recent research indicates that AOA is more efficacious in inhibiting the M-A shuttle, requiring only 0.1 mM for AAT inhibition *in vivo *[30].

**Figure 3:**
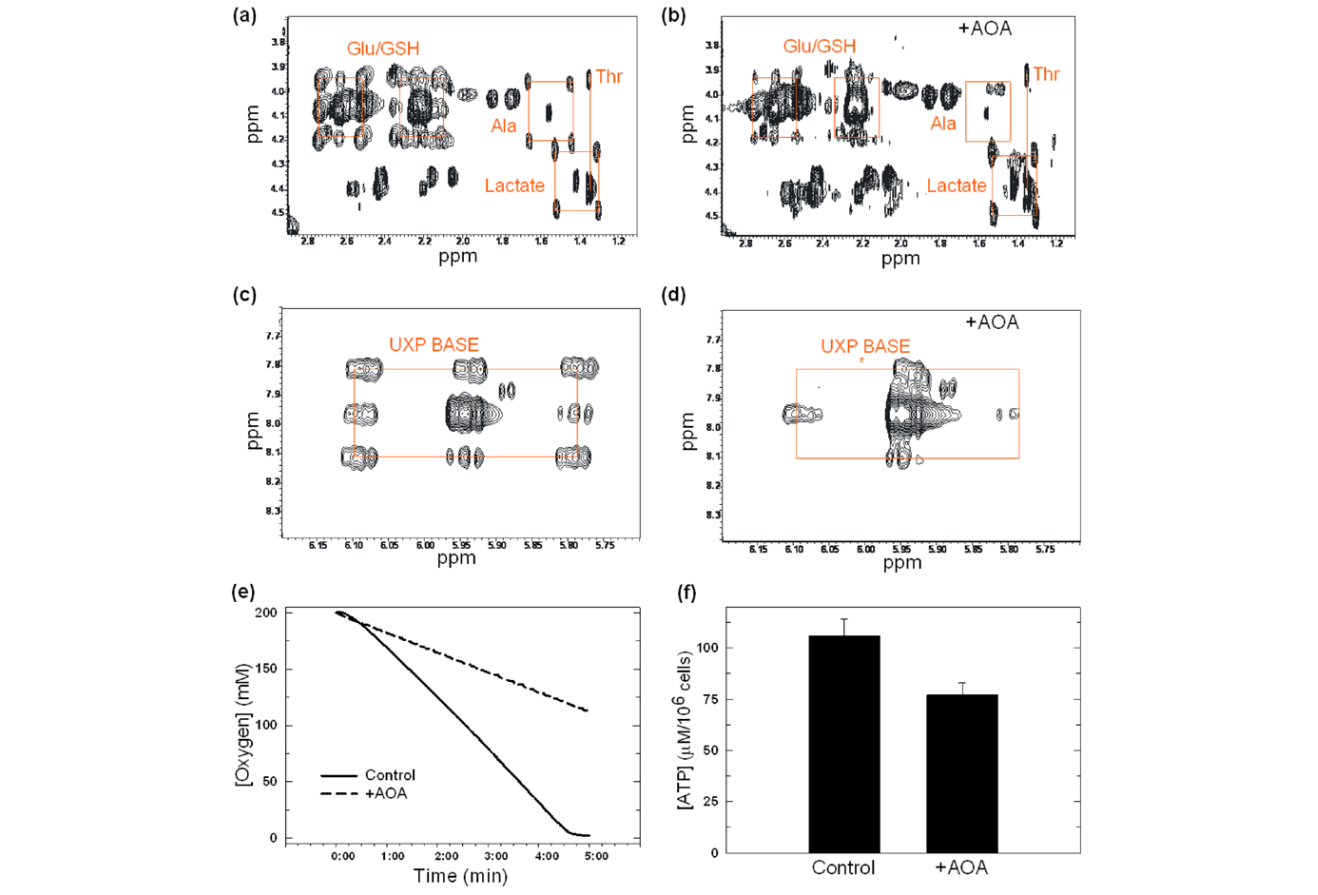
**Amino oxyacetate (AOA) inhibits the conversion of glucose into several products of the tricarboxylic acid cycle, oxygen consumption and intracellular ATP**. MDA-MB-231 cells were grown in medium containing 1 g/L [U-^13^C_6_]-glucose and exposed to either (a, c) PBS or (b, d)100 μM AOA. After 48 hours, live cells were washed, extracted with 10% trichloracetic acid (TCA), lyophilised and dissolved in 100% heavy water. The spectrum was recorded at 14.1 T with a recycle time of five seconds under standardised conditions of acquisition. ^13^C-isotopomers were quantified by indirect detection of protons in the 2D-NMR total correlation spectroscopy (TOCSY) spectrum. The four peaks representing the ^13^C-labelled glutamate/glutathione (Glu/GSH), alanine (Ala) and (a, b) lactate and (c, d) uridine bases are highlighted with red squares. Cells were cultured in the presence or absence of 100 μM AOA for 24 hours and then (e) oxygen consumption was measured using an oxygen meter and (f) ATP concentration was determined with a luminometer as detailed. ATP standard curves were run concurrently. Data are plotted as the mean ± standard deviation.

We confirmed that AOA inhibited recombinant human cytoplasmic AAT (IC_50 _= 515 ± 35 μM) and then examined the effect of 100 μM AOA on the fate of uniformly labelled [U-^13^C_6_]-glucose using 2D-NMR-TOCSY analyses. Figures 3a and 3b demonstrate the regions of the TOCSY spectra of untreated and AOA-treated MDA-MB-231 cells that contain the glutamate/glutathione, lactate and alanine peaks. The effects of AOA on glucose metabolism mirrored those observed after oxamate exposure. We observed a reduction in the ^13^C-labelled glutamate/glutathione satellite peaks and no decrease in ^13^C incorporation into lactate after exposure to AOA (Figure 3b). We also found that the uridine satellite peaks were completely abrogated by AOA treatment suggesting that the ^13^carbons are not being incorporated into uridine (Figure 3d).

Taken together, these data indicate that exposure of MDA-MB-231 cells to AOA, like oxamate, inhibits tricarboxylic acid cycling. We were surprised by the observed reduction in alanine by AOA (Figure 3b) and can only speculate that increased cytoplasmic NADH may promote conversion of pyruvate to lactate by LDH and thus reduce available substrate for alanine (i.e. pyruvate). Similar to oxamate exposure, AOA inhibited oxygen consumption, reduced the steady-state concentration of ATP after 24 hours (Figures 3e and 3f) and decreased the NAD+/NADH ratio of MDA-MB-231 (untreated, NAD+/NADH = 1.29; + 100 μM AOA, NAD+/NADH = 1.01).

### Oxamate and AOA suppress MDA-MB-231 breast adenocarcinoma growth

**Figure 4:**
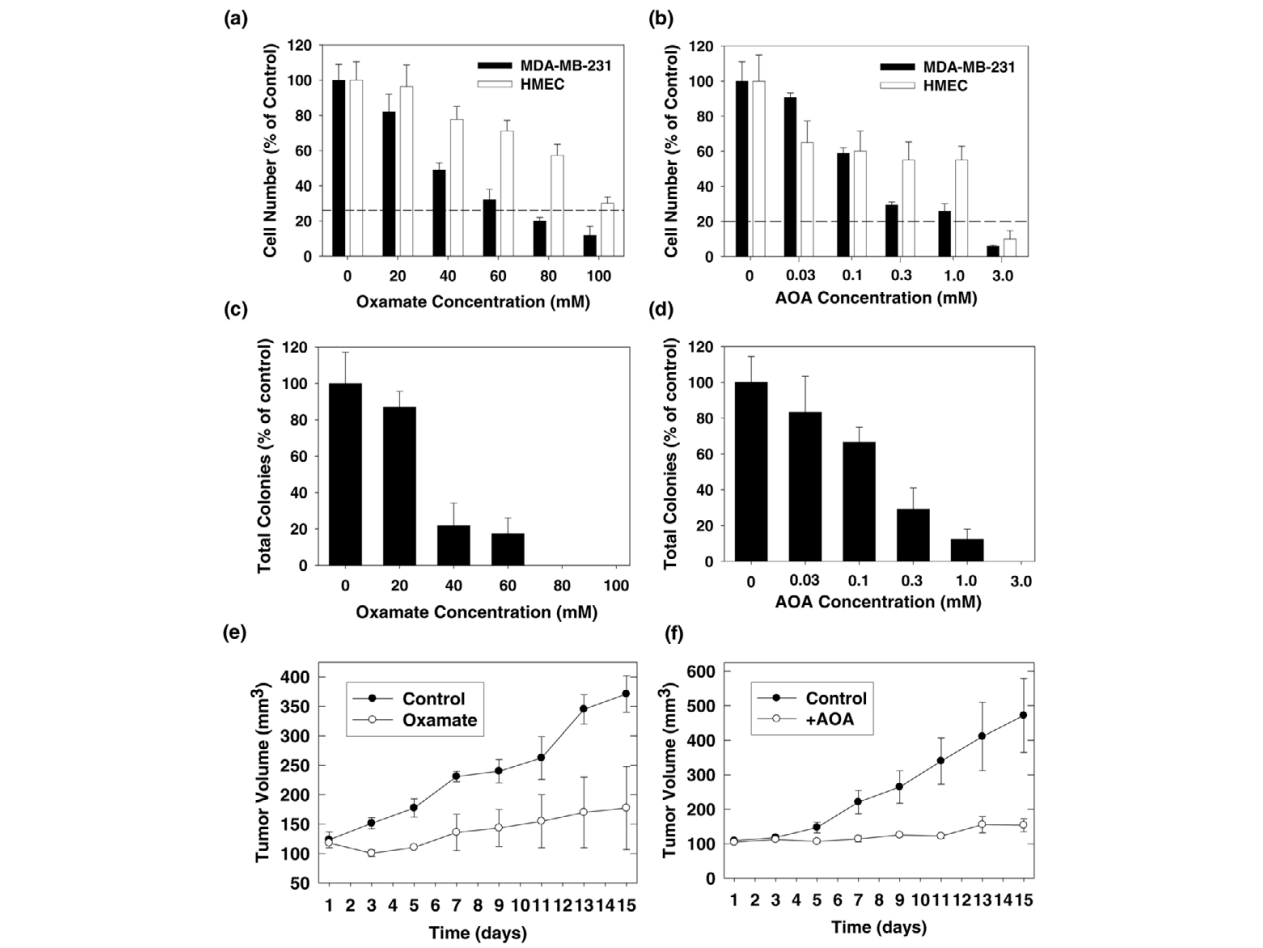
**Oxamate and amino oxyacetate (AOA) are selectively toxic to MDA-MB-231 transformed human mammary epithelial cells (HMEC) and suppress the growth of established breast tumour xenografts *in vivo***. Growth inhibition studies were performed as described. MDA-MB-231 cells or HMEC were cultured in identical media for 48 hours in the absence or presence of the indicated concentrations of (a) oxamate or (b) AOA. Cells were exposed to trypan blue and enumerated using light microscopy (dashed line indicates percentage control at time of plating). MDA-MB-231 cells at a concentration of 2.5 × 10^4 ^were plated on 6 cm dishes containing Dulbecco's Modified Eagle's Medium (DMEM) with 0.6% agar in the absence or presence of the indicated concentrations of (c) oxamate or (d) AOA. Cells were fed with 0.2% agar in media containing oxamate every three to five days. After 28 days, soft agar colonies were enumerated and expressed as a percentage of control. MDA-MB-231 xenografts were initiated as described. Tumours were measured daily using blunt end Vernier calipers, and mice with established tumours (130 to 190 mg) were blindly randomised into either PBS control (●) or treatment (○) groups. Experimental mice were weighed, and given daily intraperitoneal injections of either PBS or (e) 15 mg oxamate or (f) 0.2 mg AOA. Data are plotted as the mean ± standard deviation.

We examined the effects of several concentrations of oxamate or AOA on the proliferation of MDA-MB-231 human breast adenocarcinoma cells relative to normal HMECs. Figures 4a and 4b demonstrate that exposure to either oxamate or AOA causes a selective inhibition of growth of MDA-MB-231 transformed mammary epithelial cells when compared with HMECs. Neoplastic transformation of mammary epithelial cells confers the ability for anchorage-independent growth. We cultivated colonies of MDA-MB-231 cells in soft agar in the presence of several concentrations of oxamate or AOA. We found that exposure to oxamate or AOA inhibits soft agar colony formation at concentrations that closely mirror the inhibition of anchorage-dependent proliferation (Figures 4c and 4d). We next examined the effect of intraperitoneal administration of oxamate or AOA on the growth of established MDA-MB-231 breast tumours in athymic mice. MDA-MB-231 cells were injected subcutaneously into the flanks of groups of BALB/c athymic female mice and, after the tumours had grown to about 120 mg, the mice were randomised to receive a control injection of PBS, 15 mg oxamate or 0.2 mg AOA in PBS daily for 14 days. We found that both oxamate and AOA suppressed the growth of MDA-MB-231 tumours within 48 hours of treatment initiation (Figures 4e and 4f). Taken together, these data indicate that transformed mammary epithelial cells may be selectively sensitive to AAT inhibition.

### AAT is required for breast cancer cell proliferation

**Figure 5:**
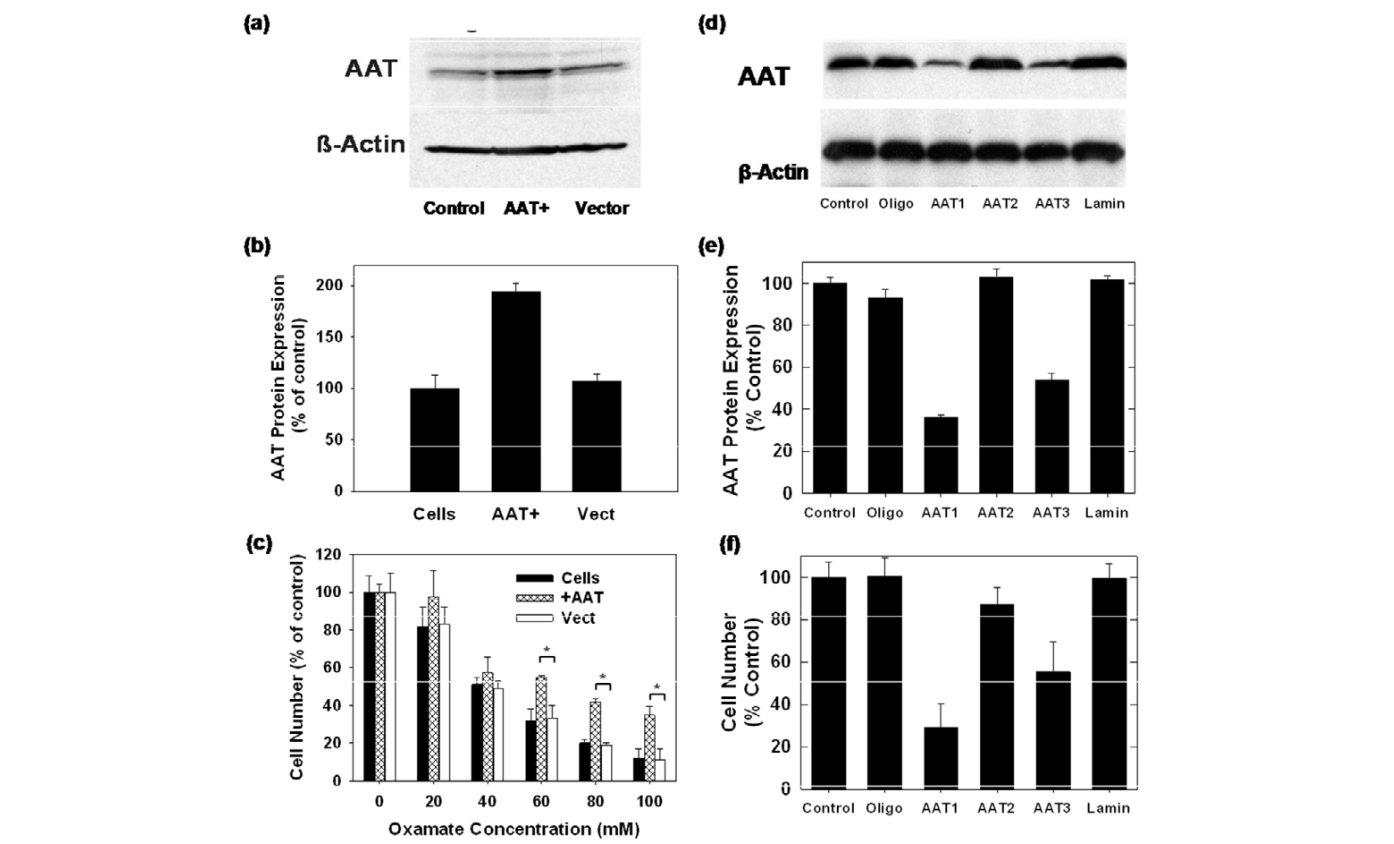
**Over-expression of aspartate aminotransferase (AAT) in MDA-MB-231 breast adenocarcinoma cells confers resistance to oxamate and AAT-specific siRNA suppresses MDA-MB-231 cell proliferation**. (a) MDA-MB-231 cells were stably transfected with AAT or empty vector and examined for AAT expression by Western blot analysis. (b) Densitometry of Western blot after over-expression of AAT. (c) Cells were incubated with the indicated concentrations of oxamate, and viable cells were counted after 48 hours. Control cells containing an empty vector were similarly examined. *p < 0.01 represents statistical difference between control and oxamate-treated samples. (d) MDA-MB-231 cells were untreated (control), sham-transfected with Oligofectamine alone (oligo) or transfected with AAT-specific siRNA molecules (AAT1-3) or lamin-specific control siRNA (Lamin) and examined for AAT expression by Western blot analysis. (e) Densitometry of Western blot after siRNA transfection. (f) Viable cells were counted 48 hours after siRNA transfection. Data are plotted as the mean ± standard deviation.

In order to directly address the hypothesis that AAT inhibition is required for the cytostatic effects of oxamate, we stably expressed AAT in MDA-MB-231 cells and examined these cells for cytostatic sensitivity to oxamate. We confirmed increased AAT expression by Western blot analysis (Figures 5a and 5b) and found that the over-expression of AAT conferred resistance to the cytostatic effects of oxamate (Figure 5c; IC_50 _values = MDA-MB-231 cells, 39 ± 6.0 mM; MDA-MB-231/+AAT cells, 68 ± 5.6 mM; MDA-MB-231/vector control, 41 ± 6.2 mM). In order to directly examine the requirement of AAT for MDA-MB-231 cell proliferation, we transfected the cells with three separate AAT-specific siRNA species (AAT1-3) and confirmed that AAT1 and AAT3 selectively inhibit AAT protein expression relative to β-Actin (Figures 5d and 5e). Selective AAT inhibition caused a marked reduction in cell proliferation as determined after 48 hours of transfection (Figure 5f). Collectively, these data provide support for the role of AAT as an essential metabolic target of oxamate and as a potential target for the development of anti-neoplastic agents.

## Discussion

We have demonstrated that oxamate and AOA selectively inhibit the proliferation of transformed breast adenocarcinoma cells *in vitro *and suppress the growth of established breast adenocarcinoma xenografts in athymic mice. Although we confirmed that oxamate inhibited recombinant LDH-A, we also found that oxamate decreased the activity of AAT, a key enzyme required for the malate-aspartate NADH shuttle and an enzymatic target of AOA. We found that oxamate reduced the flux of glucose-derived carbons into several mitochondrial tricarboxylic acid cycle products and reduced oxygen consumption but had little effect on the glycolytic flux into lactate. Importantly, we established the requirement of AAT for breast adenocarcinoma cell proliferation by finding that ectopic expression of AAT in MDA-MB-231 cells confers resistance to the anti-proliferative effects of oxamate, and that AAT-specific siRNA molecules decrease MDA-MB-231 growth *in vitro*. Taken together, these data indicate that AAT may be a novel target for the development of anti-breast cancer agents.

In order to enable untethered flux at multiple energetic and anabolic enzymes, NADH production must be balanced with NADH oxidation to NAD+. Although mitochondrial NADH can be directly oxidised in the electron transport chain, cytoplasmic NADH cannot directly traverse the mitochondrial membrane. Three NADH shuttle systems operate to transfer electrons from cytoplasmic NADH to mitochondrial NAD+: the α-glycerophosphate shuttle, fatty acid shuttle and the malate-aspartate shuttle [17-19,33,34]. Cytoplasmic AAT and mitochondrial AAT function in tandem with malate dehydrogenase to cycle the malate-aspartate shuttle. Specifically, cytoplasmic malate can enter the mitochondria via a malate-α-ketoglutarate antiporter and be converted to oxaloacetate by malate dehydrogenase, resulting in the reduction of mitochondrial NAD+ to NADH. Oxaloacetate cannot traverse the mitochondrial membrane but is converted by mitochondrial AAT into aspartate, which then enters the cytoplasm via the glutamate-aspartate antiporter and is converted by cytoplasmic AAT to oxaloacetate. This newly generated cytoplasmic oxaloacetate is then converted to malate by malate dehydrogenase, which results in the oxidation of cytoplasmic NADH and completes the shuttle cycle.

The malate-aspartate shuttle is active in the neoplastic cells of several types of tumours and is believed to account for about 20% of the total respiratory rate and to allow mitochondrial oxidation of the significant fraction of glycolytic NADH that is not oxidised by LDH-A (13 to 80% depending on the cell type [17-19,33,34]). The activities of the two essential enzymes of the malate-aspartate shuttle, AAT and glutamate dehydrogenase, are increased as a result of a combination of H-ras and carcinogen (diethylnitrosamine) induced transformation *in vivo *[33]. We speculate that the observed reduction in mitochondrial metabolism by oxamate and the reliance of the cytostatic effects of oxamate on AAT expression may be related to an essential requirement for this shuttle to be at least minimally active in rapidly dividing breast cancer cells. LDH activity has previously been found to account for the majority of the NADH-oxidising activity when compared with the malate-aspartate shuttle in transformed cells [34]. Although we found that oxamate inhibits both recombinant LDH-A and AAT, the NMR-TOCSY spectra presented herein suggest that flux through AAT may be markedly more sensitive to pharmacological inhibition by oxamate *in situ*. These data are surprising since oxamate was found to be a far more potent inhibitor of LDH-A than AAT (Figure 1). We can only speculate that the intracellular compartmentalisation of oxamate relative to its enzyme targets, LDH-A and AAT, or the intracellular equilibrium between the substrates and products of these two enzymes favours the *in situ *inhibition of AAT rather than LDH-A.

In human tissues, both cytoplasmic AAT and mitochondrial AAT proteins consist of a compartment-specific homodimer that are encoded by separate genes, GOT1 and GOT2, respectively. Although the crystal structures of the human cytoplasmic AAT and the mitochondrial AAT isoforms have not been solved, the structures of AAT from several other species have been determined, including *E. coli *[35-38], the cytosolic and mitochondrial isoforms from chickens [35-42] and the cytosolic form from pigs [43]. Analysis of these crystal structures using a least-squares minimum overlay found that the proteins, regardless of origin, have an identical peptide fold and active site residues [44]. Given the high residue sequence homology between human and pig cytoplasmic AAT, the tertiary structure of human AAT should be easily deducible via computational modelling. Based on this homology model, small molecule inhibitors of AAT then can be identified through the use of virtual screening, a computational technique that can prescreen vast databases of small molecule structures against a three-dimensional structure to see which of these dock into a chosen substrate-binding site. Accordingly, we predict that AAT may be a 'druggable' metabolic target for the development of anti-breast cancer agents.

## Conclusions

Taken together, these observations support an examination of the role of AAT in the neoplastic metabolism, transformation and growth of mammary epithelial cells. Future studies will be directed at generating a cre-lox heterozygous knockout of AAT as well as examining the relative effects of lentivirus-driven small hairpin RNA silencing of AAT on normal and transformed mammary epithelial cells. Importantly, heterozygous genomic deletions of several metabolic enzymes that are essential for neoplastic transformation have been found not to result in developmental or growth abnormalities [45-48], and we postulate that certain metabolic enzymes such as AAT may prove to be the Achilles' heel of breast cancer cells.

## Abbreviations

AAT: aspartate aminotransferase; AOA: amino oxyacetate; BSA: bovine serum albumin; EDTA: ethylenediaminetetraacetic acid; FCS: fetal calf serum; HIF: hypoxia inducible factor; HMEC: human mammary epithelial cells; IRES: internal ribosome entry site; LDH: lactate dehydrogenase; NAD+: oxidised form of nicotinamide adenine dinucleotide; NADH: reduced form of nicotinamide adenine dinucleotide; NMR: nuclear magnetic resonance; PBS: phosphate buffered saline; PCR: polymerase chain reaction; PET: positron-emission tomographic; siRNA: small interfering RNA; TOCSY: total correlation spectroscopy.

## Competing interests

The authors declare that they have no competing interests.

## Authors' contributions

JT conducted the majority of experiments and drafted the manuscript. KN aided in intellectual design of the experiments, assisted in the mouse experiments and provided aid in Western blot analysis. BFC analysed the effects of AOA on AAT. AL and SA conducted the NMR analyses. AS purified both the human recombinant LDH-A and human recombinant AAT. ST analysed the effects of AOA and oxamate on Alamar Blue oxidation in MDA-MB-231 cells. JWE assisted with the direction and the critical interpretation of the results related to oxygen consumption, NADH and ATP assays. JC conceived and directed the experiments.
